# Primary mucinous carcinoma of the skin

**DOI:** 10.1002/ccr3.7968

**Published:** 2023-09-25

**Authors:** Takayuki Yamada

**Affiliations:** ^1^ Asunaro Clinic Takasaki Japan

**Keywords:** head and neck neoplasms, margins of excision, mucinous carcinoma, skin neoplasms

## Abstract

**Key Clinical Message:**

Mucinous carcinoma of the skin is clinically characterized by good mobility, slow growth, a macroscopically smooth surface, no easy contact bleeding, and no internal flow on color Doppler; it is thus difficult to distinguish from benign tumors.

**Abstract:**

A 58‐year‐old man presented to our clinic complaining of a right cheek induration, growing over the previous 6 months. The tumor surface was smooth, pink, did not bleed easily, and approximately 10 mm in size with good mobility. Ultrasonography revealed a well‐circumscribed hypoechoic homogenous tumor with posterior acoustic enhancement; color Doppler displayed no internal flow. Preoperative diagnosis was intradermal type nevus or dermatofibroma. An excisional biopsy was performed under local anesthesia. The biopsy specimen unexpectedly showed tumor cells with an epithelial alveolar configuration floating in a mucin lake. The tumor cells contained enlarged nuclei and formed atypical columnar epithelium, arranged in fused, honeycomb‐like glandular ducts, indicating mucinous carcinoma.

## Graphical abstract

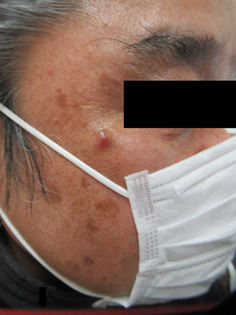

A 58‐year‐old man presented to our clinic complaining of a right cheek induration that had been growing over the past 6 months. The tumor surface was smooth, pink in color, did not bleed easily, and was approximately 10 mm in size with good mobility (Figure 1 A1, close‐up A2). Ultrasonography revealed a well‐circumscribed hypoechoic homogeneous tumor with posterior acoustic enhancement, and color Doppler displayed no internal flow (Figure 1B). Preoperative diagnosis was intradermal type nevus or dermatofibroma. An excisional biopsy was performed under local anesthesia. The biopsy specimen unexpectedly showed tumor cells with an epithelial alveolar configuration floating in a mucin lake. The tumor cells contained enlarged nuclei and formed an atypical columnar epithelium, arranged in fused, honeycomb‐like glandular ducts (Figure 1 C1, C2). These findings mark the tumor as a mucinous carcinoma. Immunohistochemical profiling revealed that the tumor cells were positive for cytokeratin‐7 (CK7; Figure 1D) and negative for CK20 (Figure 1E), indicating that this tumor originated from a primary cutaneous lesion.^1^ Surgical margins were positive for cancer cells; thus, an additional wide resection of the tumor was performed under general anesthesia. Upper gastrointestinal scope examination and chest, abdominal, and pelvic computed tomogram revealed no further lesions, leading to the diagnosis of primary mucinous carcinoma of the skin.

**FIGURE 1:**
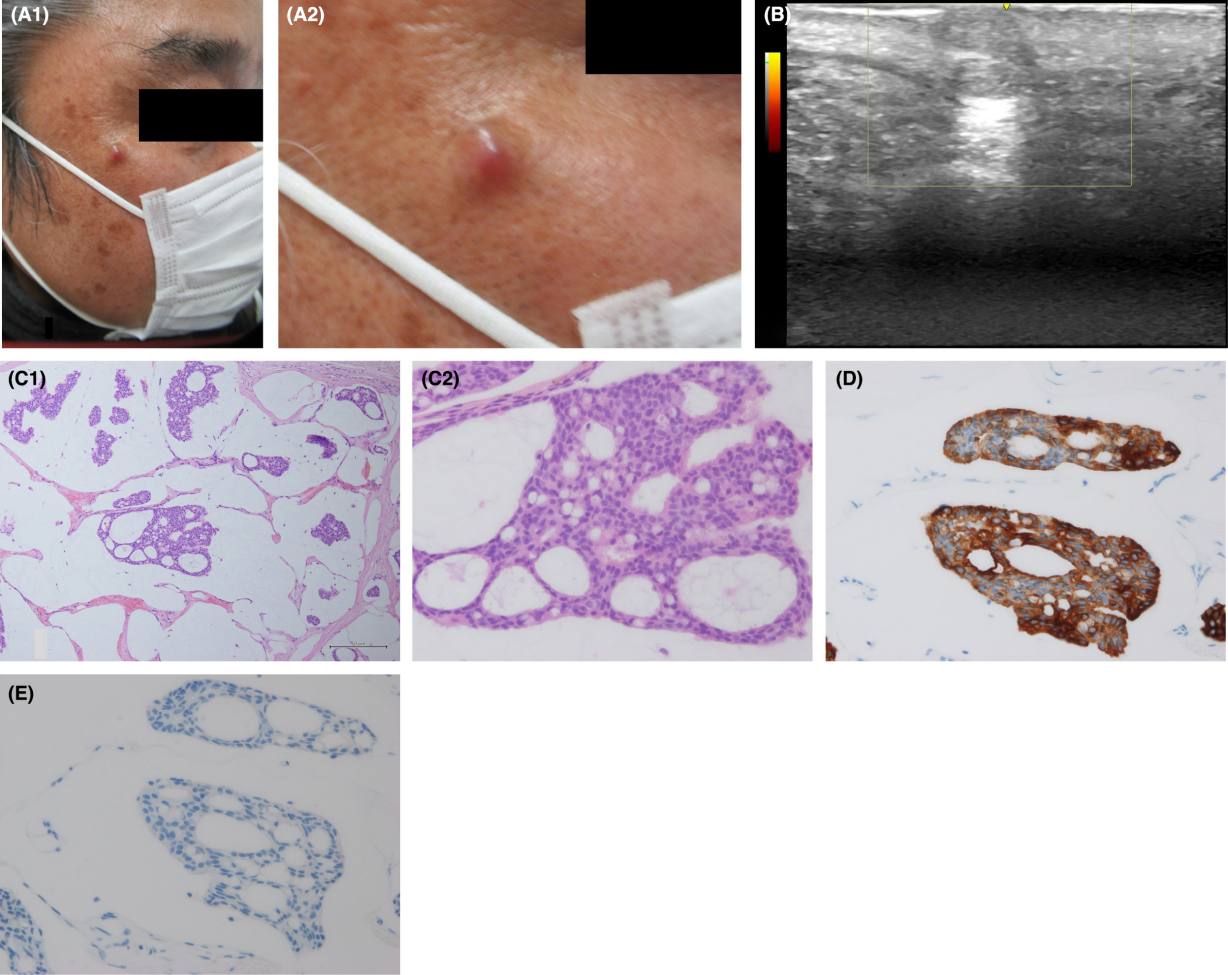
(A1): The tumor had a smooth surface, was pink in color, did not bleed easily, and was approximately 10 mm in size with good mobility. (A2): Close‐up of the tumor revealing pink color and sharp border. (B): Ultrasonography revealing a well‐circumscribed hypoechoic homogeneous tumor with posterior acoustic enhancement, and color Doppler displaying no internal flow. (C1): The biopsy specimen unexpectedly showing tumor cells with an epithelial alveolar configuration floating in a mucin lake. (C2): A higher magnification showing atypical columnar epithelial cells with enlarged nuclei forming fused glandular ducts. (D): Immunohistochemical profiling positive for cytokeratin‐7. (E): Immunohistochemical profiling negative for cytokeratin‐20.

Mucinous carcinoma of the skin is a rare tumor that is difficult to diagnose preoperatively owing to clinical characteristics that generally indicate a benign tumor, including good mobility, slow growth, a macroscopically smooth surface, no easy contact bleeding, and no internal flow on color Doppler. Complete surgical resection is the gold‐standard treatment for this tumor. Prognosis is generally good; however, an increased rate of local recurrence or distant metastasis is associated with simple local excision only, tumor size greater than 15 mm in diameter because of delayed diagnosis or lack of awareness regarding skin cancer, a lesion located on the trunk, and no extended follow‐up with the treating physician.^2^ However, wide excisions of the tumors, particularly when occurring on the face, can result in poor cosmetic outcomes. Thus, it is recommended to first obtain a pathological diagnosis and confirm the margins from an excisional biopsy before proceeding with any additional wide excisions.

In conclusion, mucinous carcinoma of the skin is clinically characterized by good mobility, slow growth, a macroscopically smooth surface, no easy contact bleeding, and no internal flow on color Doppler; it is thus difficult to distinguish from benign tumors.

## AUTHOR CONTRIBUTIONS

**Takayuki Yamada:** Supervision; visualization; writing – original draft; writing – review and editing.

## FUNDING INFORMATION

No funding was received for this study.

## CONFLICT OF INTEREST STATEMENT

The author declares no conflicts of interest for this article.

## ETHICS STATEMENT

Ethics approval was not required for this study.

## CONSENT

The patient provided written informed consent for publication.

## Data Availability

Data are openly available in a public repository that issues datasets with DOIs.

## References

[1] Fin A , D'Alì L , Mura S , et al. Primary cutaneous mucinous carcinoma of the chin: report of a case. Indian J Pathol Microbiol. 2019;62(1):173‐174. doi:10.4103/IJPM.IJPM_136_18 30706892

[2] Kamalpour L , Brindise RT , Nodzenski M , Bach DQ , Veledar E , Alam M . Primary cutaneous mucinous carcinoma: a systematic review and meta‐analysis of outcomes after surgery. JAMA Dermatol. 2014;150(4):380‐384. doi:10.1001/jamadermatol.2013.6006 24452370

